# Interview with Michael Ristow

**DOI:** 10.18632/aging.100430

**Published:** 2012-01-01

**Interview with Michael Ristow on his highly cited paper:** Ristow M, Zarse K, Oberbach A, Klöting N, Birringer M, Kiehntopf M, Stumvoll M, Kahn CR, Blüher M. “Antioxidants prevent health-promoting effects of physical exercise in humans”. Proc Natl Acad Sci U S A. 2009 May 26; 106(21):8665-70. By January 2012, “Antioxidants prevent health-promoting effects of physical exercise in humans” was cited more than 177 times. At the time of the interview it was 125 times so it is ranked as number 5 among 100,000 papers on aging in 2009.

Michael Ristow holds the Chair of Human Nutrition at the University of Jena, Germany since 2005. He studied medicine at the University of Bochum, Germany, and was trained to become a board-certified physician for internal medicine in Bochum and Cologne, Germany. He then was a post-doctoral scientist at the Joslin Diabetes Centre at Harvard Medical School with Dr. C. Ronald Kahn, and was appointed a group leader at the German Institute for Human Nutrition in Potsdam, Germany, thereafter. Dr. Ristow's research interests cover the role of mitochondrial (dys)function in human disease, especially type 2 diabetes and obesity as well as cancer growth. More recently, mitochondria-derived signaling molecules, including reactive oxygen species, and their role in disease prevention and extension of lifespan became the main focus of his laboratory.

**Aging: Did you expect your paper to become highly cited, or is this surprising to you?**

**MR:** I did anticipate that it might generate some interest, however the fact that it became highly cited is somewhat a surprise.

**Aging: Your paper seems to rule out the most revealing dogma in aging research. Did it cause any controversy?**

**MR:** Initially it did cause fierce controversy, especially at conferences. Meanwhile, and due to the fact that other papers with similar findings came out in parallel, it is turning into accepted fact. However I wish to emphasize that replication studies are needed for our specific experimental setup.

**Aging: Could you give us a history of the paper?**

**MR:** My lab is predominantly interested in interventions that are capable of extending lifespan. We study this mainly in C.elegans. Exercise, however, cannot be studies in C.elegans. Previously we had shown that glucose restriction can extend lifespan (PubMed ID 17908557, subsequently confirmed in PubMed IDs 19883616 and 19675139) by increasing mitochondrial metabolism, as previously shown for yeast. For glucose restriction we for the first time could show that this also increases ROS formation, and that antioxidants that interfere with ROS formation also prevent the extension of lifespan. This means that an increase in ROS is *required* for the extension of lifespan by glucose restriction, a mechanism that was named mitohormesis. Therefore, the obvious thing to analyze was whether physical exercise exerts lifespan-extending (or at least health-promoting) effects by increasing ROS formation. To study this, we closely collaborated with the clinical study centre at the University of Leipzig, Germany, with Matthias Blüher, and in the end observed the role of ROS in exercise that got eventually published.

**Aging: What are major discoveries in the field since your publication?**

**MR:** From my perspective, anti-aging research momentarily is in wider parts dealing with response to stressors, and a process named adaptive response or, in an applied setting, preconditioning. In regards to ROS as potential stressor that ultimately promotes stress resistance and lastly longevity significant evidence as emerged. In the exercise field this has been further promoted by John Holloszy, Malcom Jackson and José Vina, besides others.

**Aging: Could you give us a brief perspective on your future work?**

**MR:** We are interested in (i) the putative role of ROS signaling, also in genetically modified long-lived organisms, and (ii) the identification of compounds and micronutrients that mimic the state of exercise and/or calorie restriction despite a sedentary and calorically unrestricted lifestyle.

